# Anterior Scleral Thickness Profile in Keratoconus

**DOI:** 10.3390/life13112223

**Published:** 2023-11-19

**Authors:** Neus Burguera-Giménez, María Amparo Díez-Ajenjo, Noemí Burguera, Cristina Peris-Martínez

**Affiliations:** 1 Anterior Segment and Cornea and External Eye Diseases Unit, Foundation Ophthalmological Medical Hospital (FOM), Av. Pío Baroja, 12, E-46015 Valencia, Spain; amparo.diez@uv.es (M.A.D.-A.); cristina.peris@fom.es (C.P.-M.); 2 Department of Optics and Optometry and Vision Sciences, Physics School, University of Valencia, Dr. Moliner, 50, E-46100 Valencia, Spain; 3 Q Vision, Ophthalmology Department, Vithas Virgen del Mar Hospital, Ctra. el Mami a Viator, Km.1, E-04120 Almería, Spain; noemiburgueraid@qvision.es; 4 Surgery Department, Ophthalmology, School of Medicine, University of Valencia, Av. Blasco Ibáñez, 15, E-46010 Valencia, Spain

**Keywords:** anterior scleral thickness, keratoconus, inferior–superior asymmetry, swept-source OCT, sclera

## Abstract

Purpose: Considering that peripheral corneal thinning occurs in keratoconus (KC), the anterior scleral thickness (AST) profile was measured to compare thickness variations in healthy and KC eyes across several meridians. Methods: This cross-sectional case–control study comprised 111 eyes of 111 patients: 61 KC eyes and 50 age- and axial-length-matched healthy eyes. The AST was explored at three scleral eccentricities (1, 2, and 3 mm from the scleral spur) across four scleral zones (nasal, temporal, superior, and inferior) by using swept-source optical coherence tomography. The AST variations among eccentricities and scleral regions within and between groups were investigated. Results: The AST significantly varied with scleral eccentricity in healthy eyes over the temporal meridian (*p* = 0.009), whereas in KC eyes, this variation was observed over the nasal (*p* = 0.001), temporal (*p* = 0.029) and inferior (*p* = 0.006) meridians. The thinnest point in both groups was 2 mm posterior to the scleral spur (*p* < 0.001). The sclera was thickest over the inferior region (control 581 ± 52 μm, KC 577 ± 67 μm) and thinnest over the superior region (control 448 ± 48 μm, KC 468 ± 58 μm) in both populations (*p* < 0.001 for all eccentricities). The AST profiles were not significantly different between groups (*p* > 0.05). The inferior–superior thickness asymmetry was statistically different 2 mm posterior to the scleral spur between groups (*p* = 0.009), specifically with subclinical KC (*p* = 0.03). There is a trend where the asymmetry increases, although not significantly, with the KC degree (*p* > 0.05). Conclusions: KC eyes presented significant thickness variations among eccentricities over the paracentral sclera. Although AST profiles did not differ between groups, the inferior–superior asymmetry differences demonstrated scleral changes over the vertical meridian in KC that need further investigation.

## 1. Introduction

Keratoconus (KC) is a corneal disorder characterized by corneal ectasia, irregular astigmatism and visual acuity reduction [1]. It is traditionally defined as a non-inflammatory disease; however, currently, it can be reclassified as an inflammatory disorder given the increase in oxidative stress [2]; the elevated levels of interleukin-6 (IL-6), tumor necrosis factor-α (TNF-α) and matrix metalloproteinase (MMP)-9 in the tear films of patients with KC [3]; and the implication of the posterior segment structures in KC pathogenesis [4]. The reported prevalence and the annual incidence in the general population are rising because more advanced devices are used in clinical practice, increasing diagnostic sensitivity and the ability to identify corneal ectasia [5]. However, the KC pathophysiology is not well understood yet. It is described as a multifactorial disease associated with environmental, biomechanical, genetic, physical and biochemical disorders that modify the corneal tissue. These modifications result in epithelial structural irregularities, breaks in Bowman’s layers and stromal thinning due to an alteration in the distribution of collagen fibers [6,7].

In recent years, the scientific evidence for the peripheral cornea’s role in the disease process has been growing. Brautaset et al. [8] recently suggested that peripheral corneal thinning occurred outside the ectatic region toward the corneal limbus. Likewise, Alkanaan et al. [9] demonstrated that collagen microfibril (CF) degeneration and disturbances in proteoglycans (PGs) were higher in the peripheral than in the central stroma of KC corneas. On the other hand, several studies on the corneo-scleral shape pointed out that the level of scleral asymmetry was higher in KC compared to healthy eyes, with the scleral profile being more irregular and steeper in KC [10,11]. Moreover, Kopaeva et al. [12], who analyzed the trephination of scleral disks in ectatic corneas, concluded that the involvement of the scleral tissue in patients with true KC occurs. The sclera is a connective tissue composed of heterogeneous CFs, with the majority being type I [13], in a matrix of PGs and glycoproteins (GPs), similar to the cornea [14]. CFs play an important role in the tissue’s mechanical behavior [15,16] and provide a biomechanical monitoring measure that is effective in detecting and differentiating frustrated KC from healthy eyes [17]. Thus, considering the above, the anterior sclera could take part in the natural evolution of the disease and could be used as another tool to control the initial KC stages.

Previously, the anterior scleral thickness (AST) in KC was studied by Schlatter et al. [18], who used spectral-domain OCT (SD-OCT) with an anterior segment module to measure the AST at 2 mm from the scleral spur along diverse radial scleral meridians. Their findings suggest that the KC cornea undergoes thinning over the central and peripheral zones, but the thickness of the anterior sclera stroma is not affected. However, it has to be considered that the AST varies toward the scleral equator depending on the meridian and the scleral eccentricity [19,20,21], and the sclera presents anisotropic features according to the anatomical position [14]. Therefore, the study of the scleral thickness changes among eccentricities and meridians in KC is of particular importance to verify whether the thickness spatial profile differs from that of healthy eyes, which could help to further the understanding of KC’s irregular scleral shape reported by diverse authors [10,11] and to elucidate the role of the scleral thickness in KC disease management. Thanks to technical advances in imaging, scleral analysis using swept-source OCT (SS-OCT), which uses longer wavelengths and provides a deeper structural resolution, would improve the AST estimation in comparison to the enhanced depth imaging technique (EDI) [22] or SD-OCT [23] employed by Schlatter et al. [18].

Hence, the aim of the current study was to analyze the AST in a KC population across several scleral regions and eccentricities using SS-OCT to compare it to a healthy population and to evaluate the ocular factors linked to the AST. Analyzing the variations in the AST profile and the correlated ocular parameters may provide novel insights into KC scleral tissue.

## 2. Materials and Methods

### 2.1. Patients

A prospective non-randomized case–control study was performed at the Foundation Ophthalmological Medical Hospital (FOM, Valencia, Spain). Prior to the start, the study was approved by the Ethics Committee of FOM (V1 1-04-2019), and it adhered to the tenets of the Declaration of Helsinki. Patients were recruited from the FOM and the Faculty of Optics and Optometry at the University of Valencia (Valencia, Spain) between February 2019 and March 2020. Written informed consent was obtained from all subjects, and to prevent potential bias from arising through the choice of both eyes, only one eye was randomly selected.

All participants underwent a comprehensive ophthalmic examination prior to the study’s involvement, including refraction, corrected distance visual acuity (CDVA) using an ETDRS test (Precision vision, Woodstock, IL, USA), slit lamp biomicroscopy exam, axial length (AL) with an IOL Master 700 (Carl Zeiss Meditec, Jena, Germany) and tomography analysis with a rotating Scheimpflug camera, Pentacam HR (Oculus Optikgeräte GmbH, Wetzlar, Germany). The exploration was carried out by a skilled examiner using the same standard protocol; three measurements in random order were fulfilled by all devices and the best quality measurement was selected for further analysis according to the manufacturer’s recommendation.

The inclusion criteria for both groups were 18 years of age or above and AL between 22 mm ≤ AL ≤ 26 mm due to the relationship between AST and AL [24]. Healthy eyes should have a spherical equivalent (SE) up to ±6 diopters (D), CDVA ≥ 0.00 logMAR (20/20) and no clinically detectable signs on slit lamp that could mask topographic anomalies. KC eyes have to exhibit at least one clinical sign on the slit lamp (Fleischer ring, Vogt striae, stromal thinning or anterior stromal scar) and an asymmetric bowtie pattern in corneal topography. Patients with previous crosslinking, intracorneal ring segments, refractive surgery, any history of ocular trauma, other corneal diseases, or the use of contact lenses and topical medications at least two weeks before the examination were excluded from the study.

The study sample included healthy eyes (control group, CG), and eyes with subclinical or previously diagnosed KC (KC group, KG). The sample was stratified based on the Belin–Ambrosio enhanced ectasia display (BAD-D index) [25], which has a reliable ability to distinguish normal from KC corneas [26], and topographic keratoconus classification (TKC) from Pentacam. Each eye was classified as CG, BAD-D < 1.30 D, and KG, BAD-D ≥ 1.3 D. KG was graded in four subgroups: (1) subclinical KC: TKC = possible, BAD-D ≥ 1.3 and <3.5 and fellow eye with diagnosed KC; (2) grade 1 or mild KC: TKC = 1 or 1–2 and BAD-D ≥ 3.5 and < 5.5; (3) grade 2: TKC = 2 and BAD ≥ 5.5 and < 10; (4) moderate KC: TKC = 2–3, 3 or 3–4 and BAD-D ≥ 10. The Pentacam software (version 1.21r59) set a BAD-D cut-off value of 1.6 D and 3.0 D to distinguish between normal, subclinical and KC. However, for convenience, to have balanced KC subgroups, the cut-off was slightly modified to 1.3 D and 3.5 D. If the BAD-D index was out of the established range by more than ±0.70 D, it was considered as mandatory criteria. This value was selected considering the KC repeatability study of Kreps et al. [27]. To ascertain whether the individual in the subclinical group fulfilled the KC criteria, the ophthalmologist (CPM) confirmed that the fellow eye presented diagnosed KC and the subclinical KC eye included exhibited at least one clinical sign. To ensure that stromal scars did not affect the Pentacam measurements, all scans included had to present a quality score of ‘ok’. A small subgroup of patients with superior KC was also included. The superior KC must meet all of the inclusion criteria of the KG. In addition, maximum keratometry (Kmax), apical corneal thickness (APT), and thinnest corneal thickness (TCT) might be positioned in the superior quadrant of the topography.

### 2.2. Experimental Procedure

Anterior scleral thickness measurements were performed after the initial screening of all participants. The session was scheduled the same day in the afternoon between 3:00 p.m. and 7:00 p.m. to minimize the confounding effects of diurnal variations in AST [19]. Scleral imaging and scleral data analysis were carried out using the method previously described by Burguera-Giménez et al. [28]; however, a short summary is described below.

#### 2.2.1. Scleral Imaging

The Casia2 (SS-OCT, Tomey, Nagoya, Japan) was used to evaluate AST across four scleral regions (nasal, temporal, superior and inferior meridians). This device is based on Fourier domain technology and it uses a 1310 nm wavelength swept-source laser to capture images with a scanning speed of 50.000 A-Scan/s, 10 μm axial resolution and 30 μm transverse resolution of the ocular tissue. The scan dimensions are 16 mm (width) × 16 mm (length) × 13 mm (depth). All image acquisitions have been conducted by using anterior global scan method which consists of a radial scan of 16 cross-sectional images averaged from 128 images with a scan resolution of 800 A-scan, a resolution image of 800 × 11,000 pixel per inch (PPI) per line sampling and a depth of 6 mm. An automatic eye-tracking correction was employed by the device to track the eye in real time, providing en-face images that improve the contrast and definition scan [29]. The sequence analysis of each meridian was conducted three times in random order and following a blind technique. The observer measured the AST and was unaware of the group to which the patient belonged. The mean value of all three estimations was used for further statistical analysis.

**Figure 1:**
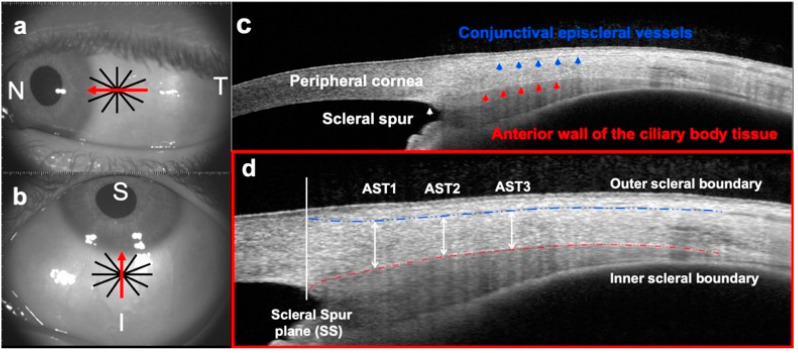
Overview of the sequential image analysis procedures implicated in the anterior scleral thickness (AST) measurement. (**a**,**b**) En-face images of anterior segment of the eye where the red arrow represents the single line passing through the scleral reflex selected of the 16-radial pattern at (**a**) horizontal (line scan of 180°) and (**b**) vertical meridian (line scan of 90°). (**c**) A B-scan of the anterior sclera where the anatomical structures are labeled (**d**) B-scan image of the anterior ocular layers to perform the consecutive thickness measurements from the scleral spur plane (SS), reference point, to the scleral equator at 1 mm (AST1), at 2 mm (AST2) and at 3 mm (AST3) where the blue line indicates the outer boundary, identified as the hypo-reflective conjunctival episcleral vessels, and the red line represents the inner boundary, identified as the hypo-reflective anterior wall of the ciliary body tissue. AST was manually measured using a caliper as the axial distance between both scleral walls. N: nasal; T: temporal; I: inferior; S: Superior.

All patients fixated on an external non-accommodative target for a few seconds. The test was placed 2 m away to control the AST modifications with accommodation [30] and about 20° out off-axis to rigorously ensure the scan over the same anterior scleral region. Accuracy attention was given to measure close to the limbus zone and to place one line from the radial scan aligned upon the sclero-corneal reflex while the examiner was looking at a real-time image of the patient’s eye on the video monitor. The horizontal line scan (180°) was selected for the nasal and temporal meridians (Figure 1a) and the vertical line (90°) for the superior and inferior meridians (Figure 1b). The OCT cross-sectional image was modified to include the iris and to distinguish all the structures (Figure 1c) in order to quantify the AST (Figure 1d). In order to expose a major scleral zone, lid retraction was manually performed without pressing the ocular globe at the inferior and vertical gaze.

#### 2.2.2. Scleral Data Analysis

The AST quantification was performed on the same device without exporting the images to maintain the resolution. For expediency, a scleral refractive index of 1.376 was assumed in all layers not considering the refractive indices variations [31] of the anterior segment structures in concordance with previous AST investigations [19,20,21,30,32,33] because the effect was expected to be minimal due to the small differences between the index assumed (1.376) and the conjunctival (1.38) and scleral (1.41) indices (mean difference = 0.004 and 0.034, respectively).

The anatomical landmarks were identified on the B-scan selected from the 2D anterior segment analysis. The location of the scleral spur (SS) was marked by the examiner as a depressed zone adjacent to the limbal region. The anterior scleral boundary was identified as the hypo-reflective conjunctival episcleral vessels and the posterior boundary as the hypo-reflective anterior wall of the ciliary body tissue (Figure 1c). The AST was quantified as the axial distance between both walls using a manual caliper which was accurately orientated and placed tangential to the reflective scleral interfaces for better precision. The SS plane was the baseline point to achieve three consecutive measurements every 1 mm (AST1, AST2, AST3) (Figure 1c). Hence, scleral eccentricity is considered as the thickness at 1 mm, 2 mm and 3 mm from the reference point (SS plane).

### 2.3. Statistical Analysis

All statistical analyses were performed using SPSS-software version-26.0 (Chicago, IL, USA) for MacOS. The normal distribution of all variables was tested by using the Kolmogorov–Smirnov test in CG and KG and the Shapiro–Wilk test in KC subgroups.

To evaluate the intra-observer variability for AST measurements, the Intraclass Correlation Coefficient (ICC) was mixed two ways with absolute agreement, and the within-subject standard deviation (S_w_) and the coefficient of variation (CoV; CoV% = (S_w_/mean)100) was calculated for each meridian and distance from the SS. A two-way repeated measures ANOVA was run to determine if there were AST variations among scleral eccentricity (i.e., between 1, 2 and 3 mm; three levels) and scleral region (i.e., between nasal, temporal, superior and inferior; four levels) and to explore the interaction between eccentricity and region in CG, KG and KC subgroups. In all cases, post hoc analysis with a Bonferroni adjustment was conducted if Mauchly’s sphericity test was not violated (*p*-value > 0.05). If not, the assumption of sphericity was not met and was calculated according to Greenhouse and Geisser [34] to correct the one-way repeated measures ANOVA. The Welch’s *t*-test was used to adjust for unequal sample sizes in KC subgroups to provide more precise outcomes. The Independent Student’s *t*-test or Mann–Whitney U test was used to contrast AST differences between CG and KG. One-way analysis of variance (ANOVA) with Tukey–Kramer’s post hoc adjustment was used for assessing the differences between control and KC subgroups in non-balanced groups. Forward stepwise multiple linear regression analysis (R^2^) was conducted in KG to test the relationship between AST and scleral eccentricity, scleral region and several ocular parameters. The level of significance considered was 0.05 (2-tailed) in all statistical tests.

## 3. Results

The study comprised 111 eyes of 111 patients aged between 18 and 55 years, 50 eyes of 50 healthy subjects and 61 eyes of 61 KC patients. Both groups were well matched in terms of age (*p* = 0.11) and AL (*p* = 0.08), whereas gender, cylinder, SE, CCT, TCT, APT, CV, BAD-D index and CDVA significantly differed between groups (*p* < 0.05). The demographic, refractive and ocular characteristics of the main groups are shown in Table 1, and those of the KC subgroups are shown in Table 2.

### 3.1. Intraobserver Agreement

The ICC values demonstrated excellent AST repeatability between sessions in all meridians and eccentricities in both groups. ICC ranged between 0.96 and 0.99 (*p* < 0.001, Tables S1 and S2 in the supplementary data, Supplementary Tables S1 and S2). The lowest ICC (0.96) was found in KG 2 mm posterior to SS in the superior meridian. The mean Sw in CG was 25 μm with CoV being generally of the order of 4–5%. The Sw in KG ranged from 21 μm to 79 μm. The greatest variability was found in the inferior and superior meridians (69–79 μm and 51–66 μm, respectively), whereas the lowest variability was in the horizontal meridian (21–26 μm); hence, the largest and lowest CoVs were observed in the vertical (11–14%) and the horizontal meridians (4%), respectively.

### 3.2. Scleral Thickness Variations

The results of the two-way repeated measures ANOVA revealed that there was significant main effect of eccentricity, CG (F (1.44, 69) = 3.7, *p* = 0.044, ηp^2^ = 0.07) and KG (F (1.5, 114) = 4.44, *p* = 0.023, ηp^2^ = 0.072), and region, CG (F (3.144) = 78.01, *p* = 0.0001, ηp^2^ = 0.619) and KG (F (3.171) = 58.40, *p* < 0.001, ηp^2^ = 0.51), on AST. Furthermore, there was a significant interaction between eccentricity and scleral region in both groups, CG (F (4.5, 215.5) = 2.60, *p* = 0.03, ηp^2^ = 0.05) and KG (F (3.75, 213.37) = 2.60, *p* < 0.001, ηp^2^ = 0.09).

#### 3.2.1. Thickness Variations across Scleral Eccentricity

**Figure 2:**
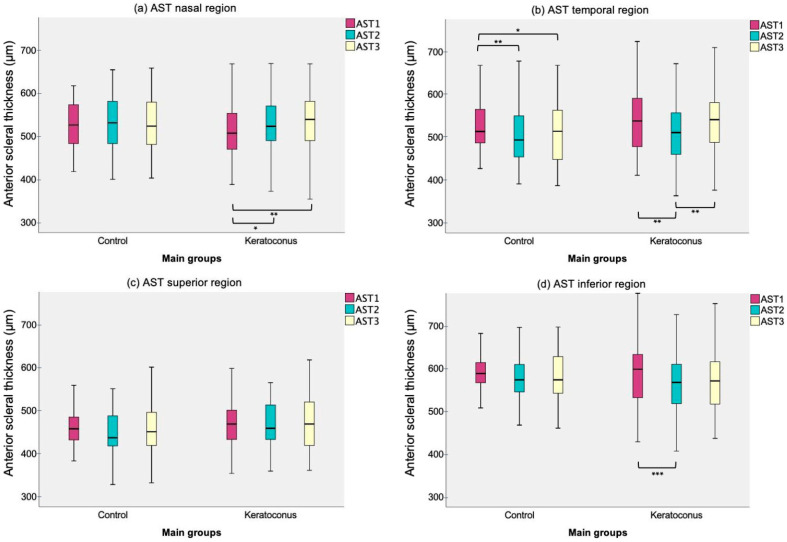
Anterior scleral thickness (AST) box plot by main groups (control and keratoconus) at each scleral eccentricity (AST1, AST2 and AST3) upon (**a**) nasal, (**b**) temporal, (**c**) superior and (**d**) inferior scleral region. Significant paired comparisons with Bonferroni post hoc adjustment among scleral eccentricity are indicated by an asterisk (*). * *p*-value less than 0.05; ** *p*-value less than 0.01; *** *p*-value less than 0.001.

**Figure 3:**
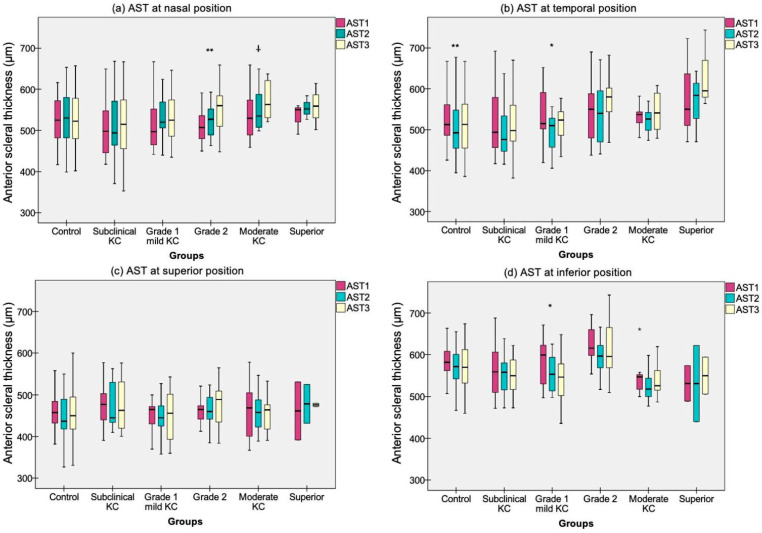
Anterior scleral thickness (AST) box plot by different grades of keratoconus (KC) over the three scleral eccentricities explored (AST1, AST2, AST3) upon (**a**) nasal, (**b**) temporal, (**c**) superior and (**d**) inferior scleral meridians. KC was stratified by using the Belin ABCD progression display [25]. Significant paired comparisons with Tukey’s post hoc adjustment among scleral eccentricity are indicated by an asterisk (*). * Statistical significance < 0.05; ** Statistical significance < 0.01; Differences that show trends to significance are indicated by a cross ^(†**)**^.

Both groups exhibited the thinnest AST 1 mm posterior to SS over nasal meridian (CG 525 ± 51 μm, KG 514 ± 61 μm) compared to the other locations, whereas toward 2 mm along the temporal (CG 497 ± 63 μm, KG 518 ± 75 μm), superior (CG 448 ± 48 μm, KG 468 ± 58 μm) and inferior regions (CG 578 ± 57 μm, KG 568 ± 64 μm). Repeated measures ANOVA revealed significant thickness changes among scleral eccentricity in healthy (*p* = 0.009) and KG (*p* = 0.029) over the temporal region, specifically in grade 1 (*p* = 0.022). However, KG also manifested AST variations upon the nasal (*p* = 0.001), in particular grade 2 (*p* = 0.016) and moderate KC (*p* = 0.05), and over the inferior region (*p* = 0.006), in particular grade 1 (*p* = 0.009). The multiple Bonferroni and Tukey’s post hoc comparisons by group and KC subgroups are displayed in Figure 2 and Figure 3, respectively.

#### 3.2.2. Thickness Variations across Scleral Region

The average AST in both groups was significantly thicker at the inferior region (CG 581 ± 52 μm, KG 577 ± 67 μm) and significantly thinner at the superior region (CG 453 ± 41 μm, KG 471 ± 60 μm) compared with that of any other meridian (*p* < 0.001, for all pairwise comparison at all locations in all groups). Repeated ANOVA measurements revealed that the amount of thickness change along the horizontal meridian was insignificant in CG, KG and independent of KC severity (mean difference (m.d.) < 20 μm across all eccentricities, *p* > 0.05) except in healthy eyes at 2 mm point (m.d. ± SD = 34 ± 72 μm, *p* < 0.0001).

### 3.3. Thickness Variations between Groups

**Figure 4:**
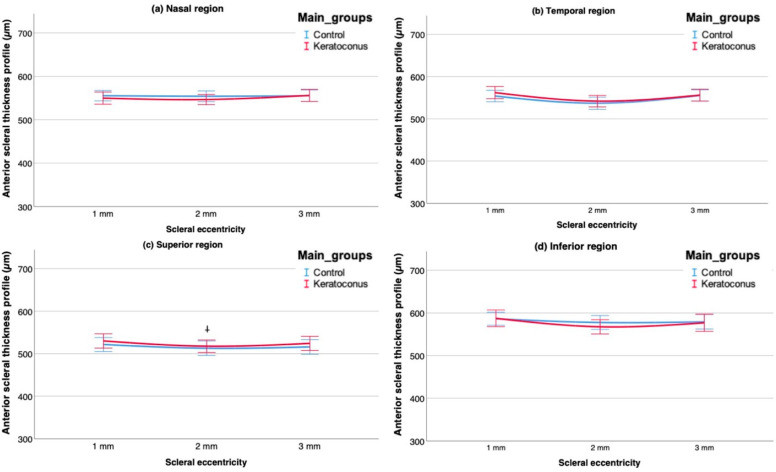
Anterior scleral thickness profile (AST) in both populations, keratoconus (KC) (red line) and control (blue line), upon (**a**) nasal, (**b**) temporal, (**c**) superior and (**d**) inferior scleral meridians. KC was stratified by using the Belin ABCD progression display [25]. The clinically relevant unpaired comparisons between groups are indicated by a cross (^†^). Error bars represent 95% of the confidence interval.

The AST profile was similar in both groups along the four scleral regions analyzed (Figure 4). However, thicker values were observed in KG over the temporal and superior regions showing a trend to significance at 2 mm in the superior meridian (*p* = 0.06). The unpaired comparisons showed no statistical differences over any eccentricity throughout meridians (*p* > 0.05 for all comparisons). The Tukey’s post hoc comparison revealed no differences between healthy and any degree of KC over each point analyzed.

**Figure 5:**
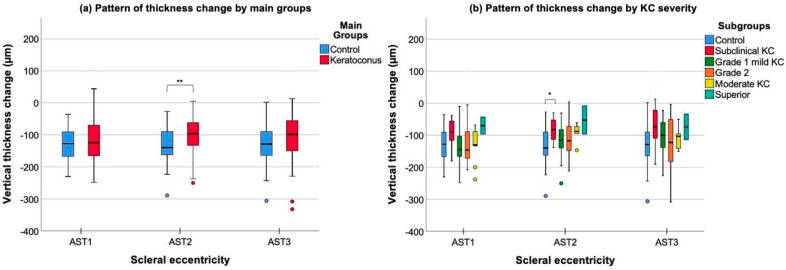
Inferior−superior thickness changes over the three scleral locations analyzed (1, 2 and 3 mm posterior to the scleral spur) by (**a**) main study groups (keratoconus (KC) and control), and by (**b**) KC subgroups. KC was stratified by using the Belin ABCD progression display [25]. The significant unpaired comparisons between groups and the significant unpaired comparisons with Tukey’s post hoc adjustment are indicated by an asterisk (*). * Statistical significance < 0.05; ** Statistical significance < 0.01.

Thickness variations along the vertical meridian (superior−inferior meridian, Figure 5a) at all eccentricities were greater in CG (m.d. ± SD = 129 ± 53 μm) than in KG (m.d. ± SD = 107 ± 53 μm) being statistically different 2 mm posterior to the SS (m.d. ± SD = 29.80 ± 11 μm, t (105) = −2.65, *p* = 0.009), specifically in subclinical KC (m.d. ± SD, healthy vs. subclinical KC = 54 ± 17 μm, F (5) = 2.50, *p* = 0.03). As the KC degree progressed, the amount of vertical thickness change tended not to increase significantly from subclinical KC to grade 3 (m.d. ± SD = 78 ± 53 μm (Subclinical KC) < 117 ± 62 μm (mild KC) < 117 ± 65 μm (moderate KC) < 123 ± 60 μm (grade 3)) regardless of the scleral eccentricity (Figure 5b).

### 3.4. Factors Influencing Scleral Thickness in Keratoconus Eyes

The multiple linear regression between AST, eccentricity, scleral region and several ocular variables (Table 3) confirmed that AST was strongly linked with the scleral region, followed by CV, SE, age, gender, TCT and CCT (adjusted R^2^ = 0.452, *p* < 0.001). The APT, AL, PIO and scleral eccentricity were not associated with AST (*p* > 0.05).

## 4. Discussion

The present study aimed to investigate if scleral thickness variations occur in KC eyes along scleral eccentricity and several meridians to compare it to a healthy population and to evaluate the linked factors with AST in individuals with corneal ectasia. In addition, KC eyes were also graded to analyze if thickness modifications diverged with KC severity.

To conduct this, in the first place, the intra-observer repeatability was verified in both groups. An excellent agreement within sessions was confirmed for AST measurements according to the results reported by previous studies in healthy eyes [19,20,32]. The CoV was lower in healthy eyes compared to KC eyes. The superior and inferior meridians in KG presented higher values of CoV which could be due to the inclusion of KC of varying degrees, the disparity in the refractive error or the relationship between AST and SE over the inferior meridian in myopic eyes described by Dhakal et al. [33].

Consistent with the majority of studies [19,20,21,30,32,33,35] in healthy eyes, our findings demonstrated that AST depends on scleral eccentricity and scleral meridian in both groups. The AST decreases significantly and gradually from the scleral spur up to 2 mm and slightly increases toward 3 mm in healthy eyes over the temporal meridian; meanwhile, in KC, temporal, nasal and inferior meridians do not show homogeneous variations with KC severity. Previous research in enucleated eyes [36,37] and in in vivo studies [19,20,21,30,33], by contrast, reported that in healthy eyes, the AST significantly decreased on moving away from limbus over all meridians. Most of these studies considered the refractive error to stratify the sample in emmetropic and myopic eyes, unlike the current work. Both groups were not well matched in terms of SE. There was a more significant diversity of refractive error in healthy than KC eyes which could justify the meridional differences. However, as Dhakal et al. [33] proved, AST profiles only differ in the inferior sclera between emmetropic and high myopic eyes (SE < −8D), which was not included in the present study. On the other hand, KC is strongly associated with compulsive eye rubbing [38] and may produce an alteration of scleral biomechanics over the inferior paracentral zone, similar to the cornea, which could explain better these thickness differences. Moreover, Kopaeva et al. [12] observed a fiber homogeneity loss in the scleral stroma of true KC. Schlatter et al. [18], who analyzed the KC scleral thickness, reported thinner values 2 mm posterior to the scleral spur. However, these results cannot be compared to our results because they were measured in the diagonal meridian and the sclera is an anisotropic tissue with variable position-dependent biomechanical features [14]. This is the first study that analyzed the scleral thickness variations in eccentricity in KC eyes and there are no previous results to compare with the current outcomes. Nonetheless, the meridional findings were not consistent amongst KC degrees, and scleral eccentricity was not associated with AST; hence, the current results need further investigation.

The outcomes showed that the sclera was significantly thicker over the inferior and thinner in the superior meridian when compared to any other meridian, and independently of the KC presence and KC severity. These findings are in agreement with several authors [19,20,21,24,33,36] who described that there is a disproportionate meridional thickness in healthy eyes produced by the asymmetrical global expansion of the ocular globe (inferior more than superior) [33], the complexities of scleral biomechanics [24] and the hypoxia episode of the unexposed sclera, which generates thickness variations [19]. Despite the fact that Schlatter et al. [18] analyzed the diagonal scleral quadrants, thicker sclera was reported in the inferotemporal (491.1 ± 48.1 μm) and inferonasal (537.5 ± 48.9 μm) meridians in contrast to the superotemporal (445.8 ± 53.1 μm) and superonasal (445.3 ± 46.7 μm) meridians. Their results also demonstrated an inferior–superior disproportionated meridional thickness in KC eyes in addition to our findings. Moreover, there was a strong goodness of fit between AST and scleral region.

Scleral thickness profile was compared between groups. There were no significant differences at any scleral point explored between healthy and KC in concordance with Schlatter et al. [18], who observed no significant scleral thinning 2 mm posterior to the scleral spur throughout meridians in keratoconic eyes. However, the change in the pattern of thickness between the inferior and superior region needs further discussion. The presence of superior–inferior asymmetry is a well established characteristic in KC corneal topography [26,27]. This asymmetry has also been described in the corneo-scleral profile by several authors [10,11,39] and most recently, the diagnostic power of vertical and horizontal thickness profiles of the corneal sublayers demonstrated the ability to distinguish between suspect KC, subclinical KC and KC [40]. Our results showed that the vertical scleral thickness change at 2 mm was significantly lower in KC, specifically between healthy and subclinical KC. Although there were not significant differences between the different KC stages, the level of superior–inferior asymmetry thickness tended to increase as the KC degree advanced over all locations. Dhaese et al. [39] demonstrated that as the cornea becomes steeper, the scleral asymmetry increases, which could support the outcomes observed over the vertical asymmetric scleral thickness. Considering that both groups exhibited similar and not statistically different AST profiles, further investigations are required with larger number of keratoconic eyes in each degree to evaluate the role of AST asymmetry, especially in subclinical KC. Moreover, it could be interesting to include parameters like the conjunctival thickness to evaluate if the steeper scleral asymmetry is explained by a conjunctival thickening. Allergic diseases such as conjunctivitis and vernal keratoconjunctivitis are strongly linked with KC presence [41], and conjunctival thickness modifications could be also taking place in KC eyes.

The current study also evidenced the significant relationship between AST, CCT, TCT and CV in KC eyes. To the best of our knowledge, this has not previously been described in the literature thus reported, and these outcomes could have important implications in future KC scleral studies. Consistent with Schlatter et al. [18] and with some other authors, in healthy eyes [20,21], males presented greater overall scleral thickness than females. In concordance with Read et al. [21], our findings demonstrated that tissue thickness varied significantly with age, with older individuals exhibiting thicker sclera. The relationship between refractive error and AST was only described by Dhakal et al. [33]; however, as our results showed in KC eyes, AST could be linked with SE in KC.

This study has limitations. Considering that there was a SE diversity between groups, the inclusion of similar SE could enhance the current outcomes but the expected effect was minimal because AST seems to be affected only in high myopes over the inferior sclera [39]. Given the similar incidence and prevalence of KC in both gender populations [1,6] and the controversial literature about the gender influence over AST [18,19,20,21,24,32,36], the gender difference observed between groups will have a small effect on the results. Since the scleral tissue surface was not parallel, Laplace, refractive indices and B-Scan tilt corrections should be applied to minimize the optical distortion [31,42]. However, Read et al. [21] quantified the thickness difference with corrected and uncorrected refractive distortions being these differences of small magnitude and suggesting minor effects on scleral thickness. Further studies including larger samples and customized software to obtain isotropic AST images will improve the accuracy of AST assessment and its objective quantification which could be biased by the manual measurement.

## 5. Conclusions

The findings of our study demonstrate that KC presented significant thickness variations among scleral eccentricity in the paracentral sclera (nasal, temporal and inferior meridians). Although AST profiles over all meridians were similar in healthy, KC and KC of varying degrees, the inferior–superior asymmetry was statistically different by 2 mm posterior to the scleral spur between KC and healthy patients, specifically with subclinical KC. These thickness differences did not significantly increase with the KC severity over all meridians. The current outcomes could contribute to developing new scleral indices that increase the diagnostic accuracy between healthy and early KC stages in conjunction with other topographic indices that need further investigation. Anterior scleral thickness could be linked with several pachymetric parameters in KC eyes.

## Figures and Tables

**Table 1:** Baseline measurements of participants by main study groups, control (CG) and keratoconus (KG) group. Categorical variables are reported as *n* (percentage) and quantitative variables as mean ± standard deviation (SD).

Parameter	Main Groups (Mean ± SD)		*p*-Value ^†^
	CG (50 Eyes/50 Patients)	KG (61 Eyes/61 Patients)	
Gender (female)	36 (72%)	28 (45.9%)	0.01 * ^
Ethnicity: Caucasian	50 (100%)	55 (90.2%)	-
Arabian	-	5 (8.2%)	
Asian	-	1(1.6%)	
Age (years)	29 ± 9.6	32.3 ± 12	0.11
SE (D)	−1 ± 1.5	−2.20 ± 2.5	0.002 ** ^‡^
Cyl (D)	−0.62 ± 0.60	−2.17 ± 2.5	<0.001 *** ^‡^
CDVA, logMAR (Snellen)	−0.05 (20/17) ± 0.06	0.09 (20/25) ± 0.18	<0.001 *** ^‡^
AL (mm)	23.7 ± 1	24.1 ± 1.15	0.08
CCT (μm)	541 ± 32.70	502 ± 37.54	0.001 ** ^†^
TCT (μm)	536 ± 31.40	481 ± 40	0.001 ** ^†^
APT (μm)	541 ± 31.40	492 ± 39.67	0.001 ** ^†^
CV (mm^3^)	60.49 ± 3.52	57.6 ± 4.32	0.001 ** ^†^
BAD-D	0.83 ± 0.60	5.42 ± 3.37	<0.001 *** ^‡^
IOP (mmHg)	14.20 ± 2.26	12.80 ± 2.25	<0.001 *** ^‡^

^(†)^ Independent Student’s *t*-test, ^(‡)^ Mann–Whitney’s U test and (^) Fisher test for unpaired samples, CG versus KG. * *p*-value < 0.05; ** *p*-value < 0.01; *** *p*-value < 0.001. SE, spherical equivalent; D, diopters; Cyl, cylinder; CDVA, corrected distance visual acuity; AL, axial length; CCT, central corneal thickness; TCT, thinnest corneal thickness; APT, apical corneal thickness; CV, corneal volume; BAD-D, Belin Ambrosio Enhance Ectasia Display Index; IOP, intraocular pressure.

**Table 2:** Baseline measurements of participants by control (CG) and keratoconus (KC) subgroups. Categorical variables are reported as *n* (percentage) and quantitative variables as mean ± standard deviation (SD).

Parameter	Main Groups (Mean ± SD)						*p*-Value
	CG (*n* = 50)	Subclinical KC (*n* = 15)	KC Grade 1 (*n* = 17)	KC Grade 2 (*n* = 17)	KC Grade 3 (*n* = 9)	Superior KC (*n* = 3)	
Gender (female)	36 (72%)	5 (33%)	12 (71%)	5 (29%)	5 (56%)	1 (33%)	0.009 ** ^
Age (years)	29 ± 9.60	35 ± 11.44	28 ± 8.72	31 ± 9.62	31 ± 15.85	41 ± 17.61	0.08 ^‡^
SE (D)	−1 ± 1.5	−2.65 ± 2.36	−1.23 ± 0.90	−2.37 ± 2.50	−3.72 ± 3.97	−0.38 ± 3.28	0.004 ** ^‡^
CDVA, logMAR (Snellen)	−0.05 ± 0.06 (20/17)	0.02 ± 0.11 (20/21)	0.14 ± 0.07 (20/28)	0.07 ± 0.07 (20/23)	0.36 ± 0.28 (20/46)	0.20 ± 0.17 (20/32)	<0.001 *** ^‡^
AL (mm)	23.7 ± 1	24.6 ± 0.89	23.6 ± 1.05	24 ± 1.23	24.6 ± 0.91	23.4 ± 1.70	0.01 * ^†^
CCT (μm)	541 ± 32.70	530 ± 33.40	502 ± 33.70	495 ± 34.32	473 ± 34.62	501 ± 31.51	<0.001 *** ^†^
TCT (μm)	536 ± 31.40	515 ± 30	487 ± 32	472 ± 33	440 ± 26	482 ± 40	<0.001 *** ^†^
APT (μm)	541 ± 31.40	524 ± 31.41	498 ± 31.52	483 ± 34.80	451 ± 25.48	500 ± 31.04	<0.001 *** ^†^
CV (mm^3^)	60.49 ± 3.52	59.19 ± 4.41	57.77 ± 3.76	56.74 ± 4.92	57.36 ± 3.63	56.53 ± 3.43	0.004 ** ^†^
BAD-D index	0.83 ± 0.60	2.24 ± 0.90	4.33 ± 1.32	6.44 ± 1.21	11.60 ± 2.79	3.10 ± 2.35	<0.001 *** ^†^
IOP (mm)	14.70 ± 2.28	13.83 ± 1.98	13.16 ± 2.07	12.38 ± 2.15	10.94 ± 2.19	13.50 ± 2.65	<0.001 *** ^‡^

^(†)^ One-way ANOVA for independent variables using Welch correction for unequal samples, ^(‡)^ Kruskal–Wallis test applying Welch correction, (^) Fisher test for unpaired samples, CG versus KC subgroups. * *p*-value < 0.05; ** *p*-value < 0.01; *** *p*-value < 0.001. SE, spherical equivalent; D, diopters; CDVA, corrected distance visual acuity; AL, axial length; CCT, central corneal thickness; TCT, thinnest corneal thickness; APT, apical corneal thickness; CV, corneal volume; BAD-D, Belin–Ambrosio Enhanced Ectasia Display Index; IOP, intraocular pressure.

**Table 3:** Results of the stepwise multiple linear regression model between anterior scleral thickness (AST) and ocular parameters in keratoconus eyes. The model summary showed an adjusted R^2^ of 45.2%.

Model Summary		R		R^2^	Adjusted R^2^	Error	Durbin-Watson	
AST		0.68		0.452	0.448	58.40	1.74	
Independent Variables			B Coefficient (Std Error)		95% Confidence Interval		*p*-Value	Importance
					Lower	Upper		
Intercept			146.31 (27.59)		92.17	200.44	0.001 ***	
Meridian	Inferior		104.57 (5.32)		94.13	115.01	0.001 ***	0.617
	Nasal		59.55 (5.26)		44.85	65.49	0.001 ***	0.617
	Temporal		59.55 (5.26)		49.23	69.87	0.001 ***	0.617
	Superior		†					0.617
CV			7.1 (0.72)		5.67	8.5	0.001 ***	0.155
SE			−5.65 (0.86)		−7.33	−3.96	0.001 ***	0.068
Age			1.16 (0.19)		0.795	1.524	0.001 ***	0.06
Gender	Male		22.56 (4.07)		14.58	30.55	0.001 ***	0.05
	Female		†					0.05
TCT			−1.34 (0.28)		−1.90	−0.79	0.001 ***	0.036
CCT			0.50 (0.21)		0.08	0.91	0.019 *	0.009
APT			0.51 (0.33)		−0.135	1.16	0.121	0.004

^†^ Reference value to compare between categorical variables. *** *p*-value < 0.001. * *p*-value < 0.05. AST: anterior scleral thickness, Std: standardized, CV: corneal volume, SE: spherical equivalent, TCT: thinnest corneal thickness, CCT: central corneal thickness, APT: apical corneal thickness.

## Data Availability

Data available on request from the corresponding author Neus Burguera-Giménez (neus.burguera@uv.es).

## Supplementary Materials

The following supporting information can be downloaded at: https://www.mdpi.com/article/10.3390/life13112223/s1, The AST was measured three consecutive times, and in order to assess the repeatability of AST measurements in both groups, the Sw, CoR, CoV and ICC were calculated. Table S1: Repeatability assessment between sessions for AST measurements in healthy eyes. Table S2: Repeatability assessment between sessions for AST measurements in KC eyes.
