# Supplementary material for: Anterior Scleral Thickness Profile in Keratoconus
Source: Life (Basel). 2023 Nov 19;13(11):2223. doi: 10.3390/life13112223 (PMC10671989; doi:10.3390/life13112223)
Supplement: Supplementary file 1 [file life-13-02223-s001.zip › life-2686187-supplementary.pdf]

Table S1 and Table S2 illustrated the results of intraobserver variability in healthy and KC eyes, respectively, as supplementary data.

Table S1. Repeatability assessment between sessions for AST measurements in healthy eyes.

Meridian	Eccentricity	Mean ± SD (μm)	*ICC*	*Sw*	CoV	p-value†
**Nasal**	**AST1**	525 ± 51	0.99	21.30	0.04	0.001***
	**AST2**	531 ± 58	0.99	23.17	0.04	0.001***
	**AST3**	532 ± 65	0.98	18.42	0.03	0.001***
**Temporal**	**AST1**	522 ± 65	0.99	30.52	0.06	0.001***
	**AST2**	497 ± 63	0.98	20.24	0.04	0.001***
	**AST3**	513 ± 71	0.98	26.13	0.05	0.001***
**Superior**	**AST1**	457 ± 43	0.97	21.02	0.04	0.001***
	**AST2**	448 ± 48	0.97	16.60	0.03	0.001***
	**AST3**	453 ± 53	0.99	26.5	0.05	0.001***
**Inferior**	**AST1**	587 ± 53	0.99	28.90	0.05	0.001***
	**AST2**	578 ± 57	0.98	31.85	0.05	0.001***
	**AST3**	580 ± 59	0.99	34.25	0.06	0.001***

*****p<0.001; † p-valor ICC, two-way mixed absolute agreement individual measurements.**

AST: anterior scleral thickness, SD: standard deviation, ICC: Intraclass Correlation Index, Sw: within-subject standard deviation, CoV: coefficient of variation

Table S2. Repeatability assessment between sessions for AST measurements in KC eyes.

Meridian	Eccentricity	Mean ± SD (μm)	*ICC*	*Sw*	CoV	p-value†
**Nasal**	**AST1**	514 ± 61	0.99	22.18	0.04	0.001***
	**AST2**	527 ± 58	0.97	23.78	0.04	0.001***
	**AST3**	537 ± 71	0.98	25.46	0.04	0.001***
**Temporal**	**AST1**	538 ± 76	0.97	25.22	0.05	0.001***
	**AST2**	518 ± 75	0.99	21.21	0.04	0.001***
	**AST3**	537 ± 75	0.99	26.31	0.05	0.001***
**Superior**	**AST1**	473 ± 67	0.98	66.77	0.14	0.001***
	**AST2**	468 ± 58	0.96	51.15	0.11	0.001***
	**AST3**	472 ± 67	0.97	58.65	0.12	0.001***
**Inferior**	**AST1**	588 ± 74	0.98	74.60	0.13	0.001***
	**AST2**	568 ± 64	0.99	67.48	0.12	0.001***
	**AST3**	577 ± 77	0.99	79.33	0.14	0.001***

*****p<0.001; † p-value ICC, two-way mixed absolute agreement individual measurements.**

AST: anterior scleral thickness, SD: standard deviation, ICC: intraclass correlation coefficient, Sw: within-subject standard deviation, CoV: coefficient of variation
